# Optimal management of recurrent and metastatic upper tract urothelial carcinoma: Implications of intensity modulated radiation therapy

**DOI:** 10.1186/s13014-022-02020-7

**Published:** 2022-03-09

**Authors:** Mi Sun Kim, Woong Sub Koom, Jae Ho Cho, Se-Young Kim, Ik Jae Lee

**Affiliations:** Department of Radiation Oncology, Yonsei Cancer Center, Yonsei University College of Medicine, 50-1 Yonsei-ro, Seodaemoon-gu, 03722 Seoul, Republic of Korea

**Keywords:** Concurrent chemotherapy, Palliative radiotherapy, PD-L1, Salvage radiotherapy, Upper tract urothelial carcinoma

## Abstract

**Background:**

Upper tract urothelial carcinoma (UTUC) is rare and the treatment for recurrent or metastatic UTUC is unclear. We evaluated the outcomes of salvage and palliative radiotherapy (RT) and prognostic factors in UTUC patients and find implications for salvage and palliative RT.

**Methods:**

Between August 2006 and February 2021, 174 patients (median age, 68 years; range, 37–90) underwent salvage and palliative RT. Disease status at RT included initially diagnosed advanced disease (n = 8, 4.6%), local recurrence only (n = 56, 32.2%), distant metastasis only (n = 59, 33.9%), and local recurrence and distant metastasis (n = 51, 29.3%). The primary tumor location included the renal pelvis (n = 87, 50%), ureter (n = 77, 44.3%), and both (n = 10, 5.7%). Radical nephroureterectomy, chemotherapy, and immunotherapy were used in 135 (77.6%), 101 (58%), and 19 (10.9%) patients, respectively. Survival outcomes and prognostic factors were analysed using Cox and logistic regression analysis.

**Results:**

Salvage RT and palliative RT was administered in 73 (42%) and 101 (58%) patients, respectively. The median radiation dose was 45 Gy (range, 15–65). Two-dimensional (2D) or three-dimensional (3D) RT and intensity modulated RT (IMRT) were used in 61 (35.1%) and 113 (64.9%) patients, respectively. The median follow-up was 7.8 months. The median duration of overall survival (OS) was 13.4 months, and the 1-year OS was 53.5%. The median progression-free survival (PFS) was 4.7 months, and the 6-month PFS was 41.9%. The 6-month infield PFS was 84%. In multivariate analysis, RT method (2D/3D vs. IMRT, p = 0.007) and RT response (p = 0.008) were independent prognostic factors for OS, and RT response correlated with PFS (p = 0.015). In subgroup analysis in patients with PD-L1 data, positive PD-L1 correlated with better PFS (p = 0.009). RT response-associated factors were concurrent chemotherapy (p = 0.03) and higher radiation dose (p = 0.034). Of 145 patients, 10 (6.9%) developed grade 3 acute or late toxicity.

**Conclusions:**

Salvage and palliative RT for UTUC are feasible and effective. Patients with RT response using IMRT may have survival benefit from salvage and palliative RT. Positive PD-L1 status might be related to radiosensitivity. High-dose radiation with concurrent chemotherapy may improve RT response.

## Introduction

Upper tract urothelial carcinoma (UTUC) is a relatively rare malignancy. UTUC accounts for 5–10% of urothelial carcinomas [1]. Patients with localized high-risk UTUC have been treated with radical nephroureterectomy (RNU) with bladder cuff excision. However, such patients often experience locoregional or distant failure after surgery alone. Recently, studies have reported the risk factors for UTUC recurrence and the need for adjuvant treatment [2].

UTUC is rare and, therefore, a standard treatment for recurrent or metastatic UTUC has not been established. The treatment of UTUC is based on the research on urothelial bladder cancer. However, the oncological outcomes with similar treatment strategies have been discordant [3]. Although UTUC and urothelial bladder cancer have the same histological type, they differ in gene alteration and clinicopathologic characteristics. In terms of stage-for-stage outcomes, the prognosis for UTUC is worse than that for urothelial bladder cancer [4].

Irrespective of the aim of radiotherapy (RT) (salvage, palliative, or adjuvant), the role of RT in UTUC has not been clearly defined. Additionally, retrospective data for RT have been conflicting; therefore, the current European Association of Urology guidelines on UTUC states that there is insufficient data regarding adjuvant RT to derive conclusions [5]. Previously used conventional radiation delivery techniques such as two-dimensional (2D) or three-dimensional (3D) RT were incapable of irradiating the tumor with a sufficient dose due to the high risk of adverse effects to the surrounding normal tissues and critical organs. Advancements in radiology and radiotherapy technologies enabled accurate targeting of the tumor and intensity modulated RT (IMRT), which protects critical normal organs and administers high-dose irradiation into the tumor and can deliver higher tumoricidal doses. Consequently, RT is believed to be a good option for recurrent or metastatic UTUC as part of multimodal treatment. However, there is relatively little literature on salvage or palliative RT in patients with recurrent or metastatic UTUC.

In this study, we aimed to evaluate the outcomes of salvage and palliative RT and prognostic factors in patients with recurrent or metastatic UTUC and find implications for salvage and palliative RT.

## Material and methods

### Patients and treatments

The Institutional Review Board at our institute approved this study and waived the requirement for patient informed consent because of the study’s retrospective nature. We retrospectively reviewed our institutional registry between August 2006 and February 2021. A total of 217 patients were treated for UTUC at our institution. We excluded 43 patients who were treated with perioperative or definitive radiotherapy; subsequently, we analysed 174 patients who received salvage or palliative RT.

UTUC was staged according to the 8th edition of American Joint Committee on Cancer staging system. Primary tumors were classified into renal pelvis and ureter tumor. Multifocal tumor was staged based on the highest T stage and/or grade. For patients who received RT for recurrent tumors, the stages at diagnosis and RT were evaluated. At our hospital, RNU and bladder cuff resection is the standard surgery for UTUC. If the surgery was difficult due to a far advanced tumor, biopsy, nephrectomy, ureter segmental resection, or lymph node dissection was performed. Chemotherapy (CTx) and immunotherapy within 2 weeks before RT were defined as concurrent treatment. RT response was evaluated using computed tomography, magnetic resonance imaging, positron emission tomography and/or whole-body bone scintigraphy. Since patients who were treated between 2006 and 2021 were included, the RT technique changed with time. Therefore, patients who were treated with various RT methods, such as 2D RT and IMRT were included in the study. In salvage RT, clinical target volume (CTV) included gross tumor volume (GTV) and potential microscopic disease extension around the macroscopically visible tumor. In palliative RT, CTV was an expansion of GTV and modified by physicians according to the patient’s symptoms. In 2D/3D RT, planning target volume (PTV) included CTV and a margin of 0.5–1 cm. In IMRT, internal target volume was used to manage the respiratory motion of the target; PTV included CTV and a margin of 0.3–0.5 cm at the discretion of the physician.

### Statistical analysis

Categorical variables were compared using chi-square test. Survival curves were plotted using the Kaplan–Meier method. The median values of the covariates except the number of metastatic organs were used as the cut-off values in prognostic factors analysis. The 75th percentile value of the number of metastatic organs was used as the cut-off value. To identify the factors related to RT response, logistic regression analysis was used. To identify the factors related to overall survival (OS), and progression-free survival (PFS), the log-rank test for univariate analysis and the Cox hazards regression model with forward: conditional method for multivariate analysis were used. Significant variables on univariate analysis were included in the multivariate analysis. We used Statistical Package for Social Sciences (SPSS), version 25.0 (SPSS Inc., Armonk, NY, USA) for statistical analyses. Statistical significance was set at P value < 0.05.

## Results

### Patients and treatment

The patient characteristics are summarised in Table 1. Most of the patients had high-grade tumor (82.8%) and stage IV disease (91.4%) when they received RT. The disease status at RT included initially diagnosed advanced tumor in 8 (4.6%) patients, local recurrence only in 56 (32.2%) patients, distant metastasis only in 59 (33.9%) patients, and local recurrence and distant metastasis in 51 (29.3%) patients. Eighty-seven (50%) patients had primary renal pelvis tumor, 77 (44.3%) patients had primary ureter tumor, and 10 (5.7%) patients had primary tumors in both the renal pelvis and ureter. Twenty-three (13.2%) patients had multifocal tumors. The median tumor size was 4.1 (range, 0.3–18) cm. Lymphovascular invasion (LVI) and perineural invasion were identified in 50 (28.7%) and 21 (12.1%) patients, respectively. Programmed death-ligand 1 (PD-L1) data was available only in 43 patients; of them, 7 (16.3%) patients tested positive for PD-L1. The median interval between the diagnosis and first recurrence was 8 (range, 1–106) months.

**Table 1:** Patient characteristics

Variables	No. (%)
Age	
Median	68 (37–90)
Sex	
Male	120 (69)
Female	54 (31)
ECOG	
0	8 (4.6)
1	132 (75.9)
2	26 (14.9)
3	8 (4.6)
Primary site	
Renal pelvis	87 (50)
Ureter	77 (44.3)
Pelvis and ureter	10 (5.7)
Multifocality	
No	151 (86.8)
Yes	23 (13.2)
Tumor size	
Median (cm)	4.1 (0.3–18.0)
Grade	
Low	8 (4.6)
High	144 (82.8)
NA	22 (12.6)
Lymphovascular invasion	
Negative	83 (47.7)
Positive	50 (28.7)
NA	41 (23.6)
Perineural invasion	
Negative	112 (64.3)
Positive	21 (12.1)
NA	41 (23.6)
PD-L1	
Negative	36 (20.7)
Positive	7 (4)
NA	131 (75.3)
Initial stage	
0a	2 (1.1)
0is	2 (1.1)
I	18 (10.3)
II	19 (11)
III	52 (29.9)
IV	77 (44.3)
NA	4 (2.3)
Present disease status	
Initially diagnosed	8 (4.6)
Local recurrence only	56 (32.2)
Distant metastasis only	59 (33.9)
Local recurrence and distant metastasis	51 (29.3)
Interval between diagnosis and first recurrence	
Median (month)	8 (1–106)
Surgery	
Radical nephroureterectomy	135 (77.6)
Ureter segmental resection/Nephrectomy	8 (4.6)
LN dissection only	1 (0.6)
Biopsy only	15 (8.6)
No	15 (8.6)
Resection margin (n = 144)	
Negative	94 (65.3)
Close	25 (17.3)
Positive	18 (12.5)
NA	7 (4.9)
Chemotherapy at radiotherapy	
Yes	101 (58)
No	73 (42)
Immunotherapy at radiotherapy	
Yes	19 (10.9)
No	155 (89.1)
Aim of radiotherapy	
Salvage	73 (42)
Palliative	101 (58)
Radiotherapy method	
2D/3D	61 (35.1)
IMRT	113 (64.9)
Total dose	
Median (Gy)	45 (range, 15–65)
Fractional dose	
Median (Gy)	2.65 (range, 1.65–20)

*NA* not available, *PD-L1* programmed death-ligand 1, *2D* 2-dimensional RT, *3D* 3-dimensional RT, *IMRT* intensity modulated radiotherapy

Initially RNU was performed in 135 (77.6%) patients. Nephrectomy, ureter segmental resection, or lymph node dissection was performed in 9 (5.2%) patients. Additionally, 15 (8.6%) patients underwent biopsy only. Of 139 patients with available resection margin status, 19 (13.7%) had positive resection margin. CTx was administered to 101 (58%) patients. Gemcitabine/cisplatin regimen (63.3%) was the commonest regimen followed by methotrexate/vinblastine/doxorubicin/cisplatin regimen (16.8%). Immunotherapy was used in heavily treated patients. Nineteen (10.9%) patients received immunotherapy. Programmed death-1 (PD-1)/PD-L1 monoclonal antibody was administered to 18 patients.

### Radiotherapy

**Fig. 1:**
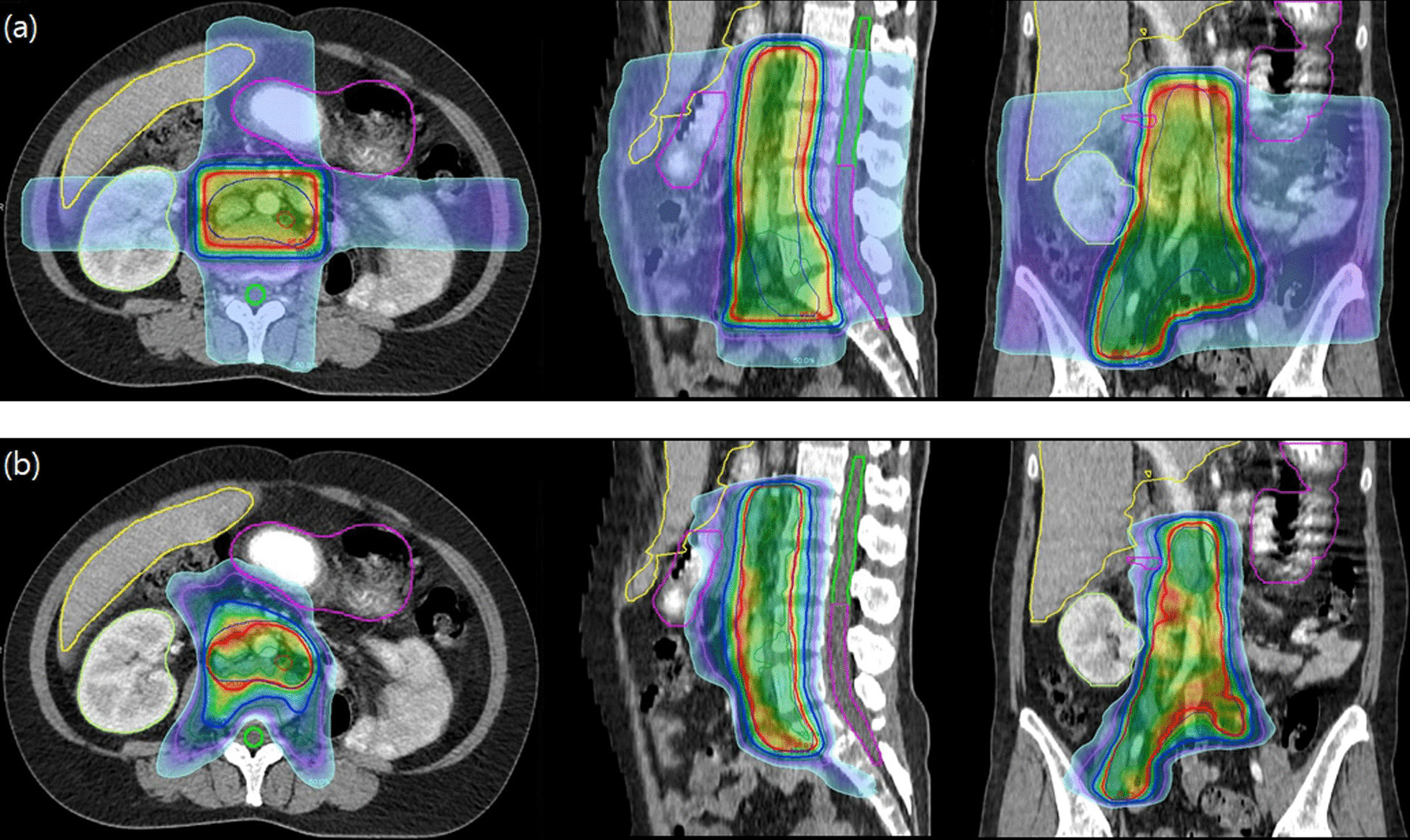
Radiotherapy plans for paraaortic–right common iliac lymph node metastasis. **a** Three-dimensional radiotherapy plan. Two anteroposterior beams and two lateral beams were used. The spinal cord, right kidney, part of stomach, liver, and adjacent small bowel were encompassed in the 50% isodose line. **b** Intensity modulated radiotherapy plan. The spinal cord, right kidney, stomach, liver, and small bowel were excluded from the 50% isodose line. Red (thick): 95% isodose line; blue (thick): 80% isodose line; sky-blue: 50% isodose line; blue (thin): planning target volume

Salvage RT and palliative RT was administered in 73 (42%) and 101 (58%) patients, respectively (Table 1). Various RT scheme were used. The median radiation dose was 45 (range, 15–65) Gy with a median fractional dose of 2.65 (range, 1.65–20) Gy. 2D or 3D RT was used in 61 (35.1%) patients and IMRT in 113 (64.9%) patients. Different RT techniques were used in different treatment periods throughout the study period (Fig. 1). During 2006–2015, 2D/3D RT was used in 80% of cases, and during 2016–2021, IMRT was used in 82.9% of cases (p < 0.001). The treatment field included the primary or recurrent tumor mass in 33 (19%) patients, tumor mass and metastatic lesions in 21 (12.1%) patients, and metastatic lesions only in 122 (70.1%) patients. The most frequently treated metastatic site included lymph nodes (70 sessions) followed by bone (58 sessions), lung (10 sessions), bladder (8 sessions), brain (5 sessions), and liver (3 sessions).

### Treatment outcomes

**Fig. 2:**
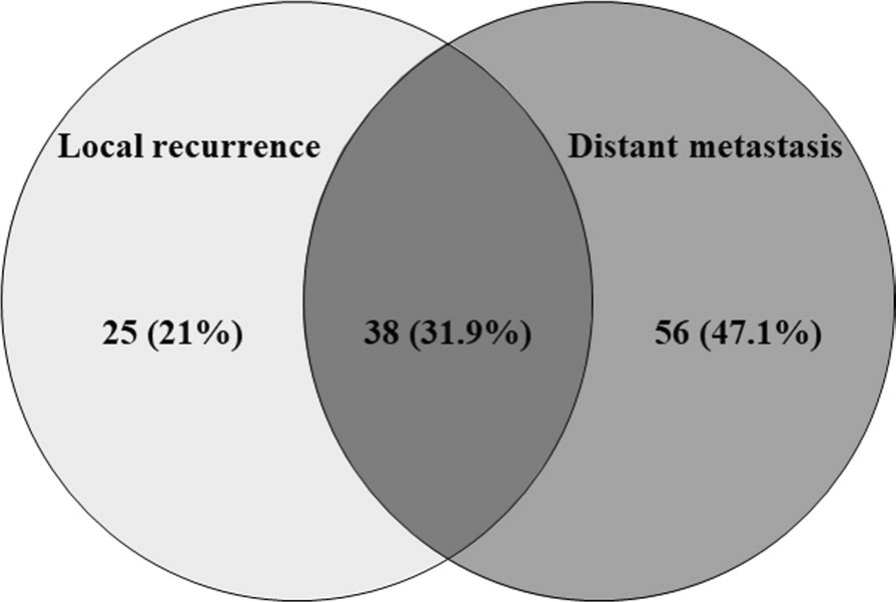
Patterns of failure. Local recurrence in 25 (21%) patients, distant metastasis in 56 (47.1%) patients, and concurrent local recurrence and distant metastasis in 38 (31.9%) patients

The median follow-up duration since RT was 7.8 months. At the last follow-up, 15 (8.6%) patients had no evidence of disease, 109 (62.6%) patients were alive with disease, and 50 (28.7%) patients had died of disease. Of 174 patients, 116 (66.7%) patients were dead, and 31 (17.8%) patients were lost to follow-up at the time of analysis. Only 137 patients were evaluable for RT response. RT responder was defined as a patient with complete remission (CR) or partial response (PR); overall, 94 patients (68.6%; CR 19.7%, PR 48.9%) were RT responders. RT non-responders with stable disease (SD) or progressive disease (PD) included 43 (31.4%; SD 24.1%, PD 7.3%) patients. The first progression after RT was found in the infield, outfield, and both infield and outfield in 7 (5.9%), 89 (74.8%), and 23 (19.3%) patients. During the follow-up, 42 (24.1%) patients had infield progression. Patterns of failure included local recurrence in 25 (21%) patients, distant metastasis in 56 (47.1%) patients, and both local recurrence and distant metastasis in 38 (31.9%) patients (Fig. 2). On univariate analysis, factors related to RT response included younger age (> 68 vs. ≤ 68 years, p = 0.012), ureter tumor (renal pelvis vs. ureter, p = 0.001), LVI (p = 0.016), concurrent CTx (p = 0.045), and higher radiation dose (≥ 45 Gy vs. < 45 Gy, p = 0.011) (Table 2). In multivariate analysis, higher radiation dose (p = 0.034) and concurrent CTx (p = 0.030) were significant prognostic factors.

**Table 2:** Prognostic factors for radiotherapy response

Variables	No. (%)	Univariate		Mulitivariate	
		OR (95% CI)	p value	OR (95% CI)	p value
Sex		0.91 (0.42–2.0)	0.813		
Male	120 (69)				
Female	54 (31)				
Age		2.68 (1.25–5.78)	0.012		
≤ 68	81 (46.6)				
> 68	93 (53.4)				
ECOG		0.84 (0.31–2.27)	0.725		
ECOG 0–1	140 (80.5)				
ECOG 2–3	34 (19.5)				
Primary location		0.25 (0.11–0.58)	0.001		
Renal pelvis	87 (53)				
Ureter	77 (47)				
Multifocality		0.62 (0.22–1.76)	0.368		
Yes	23 (13.2)				
No	151 (86.8)				
Tumor size		0.8 (0.34–1.91)	0.615		
< 4 cm	59 (46.8)				
≥ 4 cm	67 (53.2)				
Radical nephroureterectomy		0.69 (0.28–1.67)	0.406		
Yes	135 (77.6)				
No	39 (22.4)				
Resection margin		1.14 (0.34–3.87)	0.835		
Negative	95 (68.3)				
Close or positive	44 (31.7)				
Lymphovascular invasion		0.35 (0.15–0.82)	0.016		
Negative	83 (62.4)				
Positive	50 (37.6)				
Perineural invasion		0.55 (0.20–1.52)	0.245		
Negative	112 (84.2)				
Positive	21 (15.8)				
Histologic grade		0.86 (0.16–4.62)	0.856		
Low	8 (5.3)				
High	144 (94.7)				
PD-L1		0 (0–0)	0.999		
Negative	36 (83.7)				
Positive	7 (16.3)				
Concurrent chemotherapy		2.22 (1.02–4.85)	0.045	2.91 (1.11–7.65)	0.03
Yes	101 (58)				
No	73 (42)				
Interval between diagnosis to first recurrence		0.56 (0.27–1.17)	0.124		
> 8 m	81 (48.8)				
≤ 8 m	85 (51.2)				
RT methods		0.53 (0.25–1.15)	0.107		
2D/3D	61 (35.1)				
IMRT	113 (64.9)				
Radiation dose		0.38 (0.18–0.81)	0.011	2.67 (1.08–6.64)	0.034
≥ 45 Gy	81 (46.6)				
< 45 Gy	93 (53.4)				

Factors related with radiotherapy response were analyzed with logistic regression analysis

*PD-L1* programmed death-ligand 1, *IMRT* intensity modulated radiotherapy, *2D* 2-dimensional radiotherapy, 3D 3-dimensional radiotherapy

**Fig. 3:**
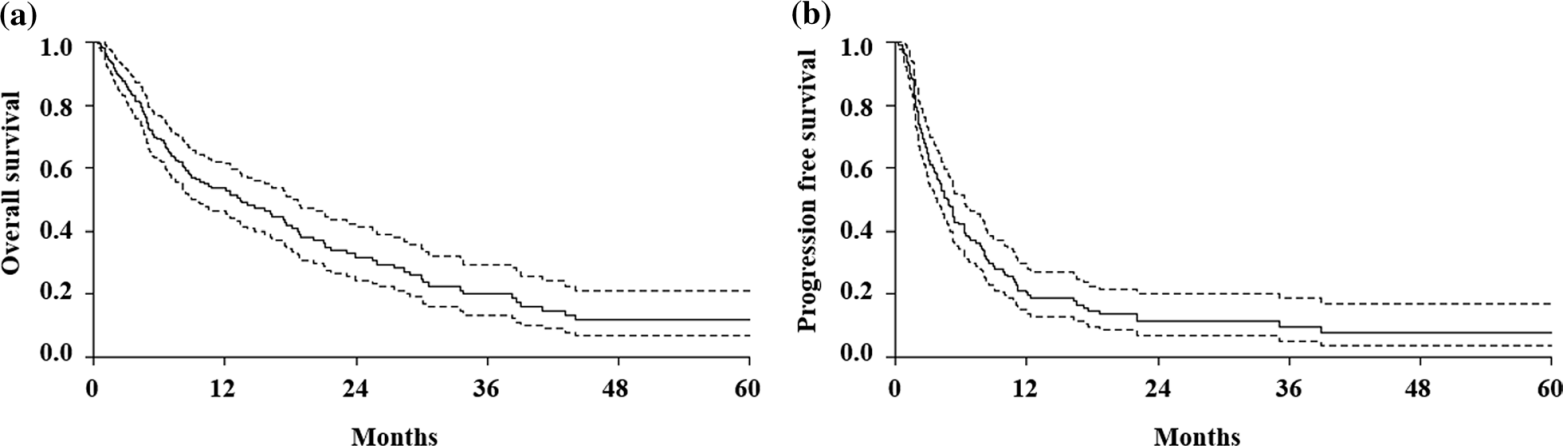
Survival outcomes. **a** Overall survival **b** Progression-free survival. Dotted lines represent 95% CIs of overall survival and progression-free survival

The median OS was 13.4 months and the 1-year OS was 53.5% (Fig. 3a). In univariate analysis, the performance status (ECOG 0–1 vs. 2–3, p < 0.001), stage at diagnosis (0-III vs. IV, p < 0.001), RNU (p = 0.015), interval between diagnosis and first recurrence (> 8 months vs. ≤ 8 months, p < 0.001), RT aim (salvage RT vs. palliative RT, p < 0.001), RT method (2D/3D vs. IMRT, p = 0.02), RT responder (p < 0.001), radiation dose (≥ 45 Gy vs. < 45 Gy, p = 0.009), and number of metastatic organs (< 3 vs. ≥ 3, p = 0.011) were associated with OS (Table 3). Tumor size (> 4 cm vs. ≤ 4 cm, p = 0.069) and negative LVI (p = 0.06) were also related with OS; however, they were not statistically significant. In multivariate analysis, RT method (p = 0.007) and RT responder (p = 0.008) were independent prognostic factors. Previously known risk factors, such as multifocal tumor, resection margin status, and histologic grade were not related to OS.

**Table 3:** Prognostic factors for overall survival and progression free survival

Variables	No. (%)	OS				PFS			
		Univariate		Mulitivariate		Univariate		Mulitivariate	
		HR (95% CI)	p value	HR (95% CI)	p value	HR (95% CI)	p value	HR (95% CI)	p value
Sex		0.98	0.909			1.04	0.833		
Male	120 (69)	(0.65–0.46)				(0.90–1.56)			
Female	54 (31)								
Age		0.83	0.331			0.65	0.023		
≤ 68	81 (46.6)	(0.58–1.20)				(0.45–0.94)			
> 68	93 (53.4)								
ECOG		2.36	< 0.001			1.55	0.081		
ECOG 0–1	140 (80.5)	(1.54–3.62)				(0.95–2.55)			
ECOG 2–3	34 (19.5)								
Primary location		1.17	0.416			1.28	0.201		
Renal pelvis	87 (53)	(0.80–1.70)				(0.88–1.86)			
Ureter	77 (47)								
Multifocality		1.1	0.855			1.06	0.831		
Yes	23 (13.2)	(0.62–1.79)				(0.62–1.80)			
No	151 (86.8)								
Stage at diagnosis		1.95	< 0.001			2.16	< 0.001	1.81	0.02
0–III	93 (54.7)	(1.35–2.84)				(1.49–3.12)		(1.10–2.99)	
IV	77 (45.3)								
Tumor size		1.52	0.069			1.5	0.062		
< 4 cm	59 (46.8)	(0.97–2.37)				(0.98–2.30)			
≥ 4 cm	67 (53.2)								
Radical nephroureterectomy		1.69	0.015			1.24	0.365		
Yes	135 (77.6)	(1.11–2.57)				(0.78–1.98)			
No	39 (22.4)								
Resection margin		1.18	0.575			1.17	0.61		
Negative	95 (68.3)	(0.66–2.10)							
Close or positive	44 (31.7)								
Lymphovascular invasion		1.53	0.06			0.483	0.001		
Yes	83 (62.4)	(0.98–2.40)				(0.32–0.74)			
No	50 (37.6)								
Perineural invasion		1.53	0.128			1.61	0.088		
Yes	112 (84.2)	(0.89–2.65)				(0.93–2.77)			
No	21 (15.8)								
Histologic grade		1.39	0.521			0.68	0.301		
Low	8 (5.3)	(0.51–3.78)				(0.33–1.41)			
High	144 (94.7)								
PD-L1		2.3	0.262			6.7	0.012		
Negative	36 (83.7)	(0.54–9.90)				(1.53–29.43)			
Positive	7 (16.3)								
Concurrent chemotherapy		1.14	0.498			0.856	0.416		
Yes	101 (58)	(0.78–1.67)				(0.59–1.25)			
No	73 (42)								
Interval between diagnosis to first recurrence		2.02	< 0.001			1.7	0.005		
> 8 m	81 (48.8)	(1.37–2.98)				(1.18–2.45)			
≤ 8 m	85 (51.2)								
RT aim		3.46	< 0.001			2.35	< 0.001		
Salvage	73 (42)	(2.31–5.20)				(1.61–3.42)			
Palliative	101 (58)								
RT methods		1.56	0.02	2.89	0.007	1.13	0.54		
2D/3D	61 (35.1)	(1.07–2.26)		(1.33–6.28)		(0.76–1.68)			
IMRT	113 (64.9)								
RT responder		3.13	< 0.001	2.76	0.008	2.55	< 0.001	1.92	0.015
Yes	93 (68.4)	(1.98–4.94)		(1.30–5.87)		(1.70–3.84)		(1.13–3.26)	
No	43 (31.6)								
Total dose		1.64	0.009			0.62	0.006		
≥ 45 Gy	81 (46.6)	(1.13–2.38)				(0.44–0.87)			
< 45 Gy	93 (53.4)								
No. of metastatic organ		2.13	0.011			1.47	0.291		
< 3	102 (86.4)	(1.19–3.81)				(0.72–2.99)			
≥ 3	16 (13.6)								

Factors related with survival were analyzed with the log-rank test for univariate analysis and the Cox hazards regression model with forward: conditional method for multivariate analysis

*RT* radiotherapy, *PD-L1* programmed death-ligand 1, *IMRT* intensity modulated radiotherapy, *2D* two-dimensional radiotherapy, *3D* three-dimensional radiotherapy

The median PFS was 4.7 months and the 6-month PFS was 41.9% (Fig. 3b). The 6-month infield PFS was 84%. In univariate analysis, younger age (p = 0.023), earlier stage at diagnosis (p < 0.001), negative LVI (p = 0.001), positive PD-L1 (p = 0.012), longer interval between diagnosis and first recurrence (p = 0.005), RT aim (salvage RT vs. palliative RT, p < 0.001), RT responder (p < 0.001), and higher radiation dose (p = 0.006) were associated with better PFS (Table 3). The tumor size was associated with PFS (p = 0.062) although it was not statistically significant. PD-L1 data was available only in 43 patients (24.7%); therefore, we excluded PD-L1 in multivariate analysis. The stage at diagnosis (p = 0.02) and RT responder (p = 0.015) remained independent predictors of PFS on multivariate analysis.

In 43 patients with available PD-L1 data, we performed subgroup analysis. All 43 patients had stage IV and high-grade tumors; therefore, we could not evaluate the effects of stage and histologic grade on PFS in them. On univariate analysis, multifocality (p = 0.01) and PD-L1 (0.012) were associated with PFS. On multivariate analysis, PD-L1 remained significantly related to PFS (p = 0.009). Performance status had borderline significance for PFS (p = 0.063).

### Toxicity

Acute toxicity data was available in 145 patients. Acute toxicity was observed in 43 (29.7%) patients. Nine (20.9%) patients had grade 3 acute toxicity, and most of them had gastrointestinal symptoms, such as anorexia and nausea. Two months after RT, one patient experienced radiation recall dermatitis with atezolizumab. Late toxicity could be evaluated in 140 patients, 12 (8.6%) of whom had late toxicity. Most common late toxicity were fatigue (41.6%) and anorexia (33.3%). One patient had grade 3 generalised weakness.

## Discussion

We retrospectively reviewed patients who received salvage or palliative RT for recurrent or metastatic UTUC. There are limitations to this study. Due to the retrospective design, heterogeneous RT scheme were used. As the aim of RT was salvage or palliative, the follow-up period was relatively short. Despite of these shortcomings, salvage and palliative RT resulted in a favourable treatment response rate of 68.6%. IMRT and RT response were beneficial for survival, and patients with positive PD-L1 had prolonged PFS in the subgroup analysis. Higher radiation dose and concurrent CTx improved the RT response. After salvage and palliative RT, no severe toxicity was observed, and the toxicity that was observed was tolerable. To our knowledge, this study is the largest report about treatment outcomes after salvage or palliative RT among patients with recurrent and metastatic UTUC.

Although there is some agreement that a satisfactory outcome cannot be obtained with surgery alone, the standard treatment for advanced UTUC or recurrent/metastatic UTUC has not been established and the research on the topic is limited. Attempts are being made to classify the molecular subtype of UTUC and apply specified treatment according to the subtype, and in the near future, individualised treatment strategies are believed to be possible. However, to date, no treatment has demonstrated a clear effect in real clinical practice. Previous studies have proposed the need for multimodal treatment [6, 7]; however, the results of studies on RT are relatively scarce. Several studies have reported on RT in a small number of patients with inoperably advanced UTUC; however, there was paucity of studies related to recurrent UTUC. One study had reported regarding the experience with salvage RT [8]. In that study, 40 patients who received RT were analysed, including 20 patients with recurrent disease. The authors reported 3-year OS of 16% and 3-year PFS of 12% in the salvage RT group. They found that higher radiation dose (≥ 50 Gy) improved the survival outcome. Similarly, we observed that higher radiation dose was positively related to the RT response. In previous studies, there was insufficient information on the radiation dose–response relationship. Various radiation dose scheme of 35–60 Gy in 1.75–2 Gy/ fraction have been reported previously [8–10].

Most previous studies focused on whether adding RT was beneficial; however, the survival benefit depending on the RT method used or the response to RT were not analysed. UTUC is a rare disease, and most studies including this study analysed patients over the long-term. Therefore, patients were treated via various techniques, from 2D RT to the latest IMRT. With 2D RT, it is impossible to selectively irradiate tumors. Therefore, it is difficult to deliver high dose due to the consideration of the organ at risk. Published studies of patients receiving RT from the 1990s to the 2000s used median dose of 45–50 Gy, which is equivalent to 55–60 Gy when converted to a biologically effective dose (BED) with α/β ratio 10. Nevertheless, several retrospective studies have demonstrated that adjuvant RT improves the survival outcomes [8–10]. Due to advancements in computer science and engineering, advanced techniques of radiation delivery have been introduced. IMRT, a novel approach in radiation planning and treatment, uses multiple photon beams with various intensities to precisely irradiate a tumor. Each beam is controlled and conforms to the shape of the tumor. IMRT permits the delivery of high dose to the tumor and minimises the dose to the critical structures around it. IMRT has been demonstrated to provide superior dose distribution compared to that provided by conventional 3D RT in various tumors [11–13]. In the treatment of prostate cancer, switching from 3D RT to IMRT can reduce the rectal volume irradiated with high dose by 25%, which results in decreased rectal toxicity [14]. Especially in patients with UTUC, IMRT can be particularly useful because the RT field often approximates the small bowel. These advances made it possible to apply high BED using hypo-fractioned RT scheme in clinical practice. Even with the same irradiation dose, the tumoricidal effect varies according to dose fractionation. For example, a dose of 60 Gy corresponds to BED 72 Gy when irradiated with conventional scheme. However, 60 Gy in 10 fractions corresponds to BED 96 Gy. Recently, advances in RT techniques took hypo-fractionated RT to the next level, stereotactic body RT and particle RT have been used. Stereotactic body RT with a dose of 50 Gy in 4 fractions (BED 112.5 Gy) has demonstrated a promising local control without severe complication [15]. Recent case report has demonstrated long-term survival outcomes after particle RT using the latest techniques with high dose of 72.6 Gy in hypo-fractionation (BED 96.6 Gy) [16].

In this study, improved survival was observed in patients treated with IMRT and in RT responders. Improved response to RT was assumed to be due to precisely irradiate the tumor and concentrate the radiation dose into the tumor in patients who underwent IMRT, resulting in the difference in survival outcomes. Large-scale analyses using the data of patients receiving RT using the latest techniques are warranted to validate our results.

Recently, a phase 3 randomised controlled trial of CTx in metastatic urothelial carcinoma has been reported [17]. The authors included approximately 25% of patients with UTUC in their study and reported a survival benefit with perioperative CTx. However, metastatic UTUC tends to have a low response rate to CTx [18]. In this study, CTx did not affect the survival outcome; however, when CTx was administered concurrently with RT, the RT response increased, which is believed to have a positive effect on survival. Other studies have also reported the effects of concurrent CTx. Huang et al. [19] administered adjuvant RT in locally advanced UTUC of stage pT3N0M0 and reported better outcomes in patients who received concurrent CTx. On comparing patients with pT3/4 and/or N + UTUC who received only adjuvant RT and those who underwent concurrent CTx and RT (CCRT), improvements in the 5-year OS and disease-specific survival were observed in patients who received CCRT [20]. A meta-analysis of perioperative treatments in 8100 patients with UTUC who underwent surgery revealed that CCRT resulted in prolonged recurrence-free survival [6].

The prognostic predictive value of PD-L1 in UTUC is controversial [21]. The cut-off level of PD-L1 varies between studies. PD-L1 positive status was observed in 3.1%–39.2% of patients with UTUC. Of 174 patients in this study, PD-L1 data were available for only 43 (24.7%). Positive PD-L1 was observed in 7 (16.3%) patients with a cut-off value of 5% and better PFS was observed in patients with positive PD-L1. Some studies have reported that overexpression of PD-L1 adversely affects survival [21]. Conversely, other studies have reported that positive PD-L1 was related with radiosensitivity in various tumors [22–25]. Using The Cancer Genome Atlas (TCGA) dataset, patients with invasive breast cancer were divided into radiosensitive and radioresistant groups [23]. The expression of CD274 mRNA, a surrogate marker for PD-L1, was significantly higher in the radiosensitive group than that in the radioresistant group, and the recurrence-free survival of the radiosensitive group was better than that in the radioresistant group. Similar results have been reported in patients with low grade glioma, glioblastoma multiforme, and head and neck cancers using TCGA dataset [22, 24, 25]. Other studies on the correlation of PD-L1 with survival outcomes following RT have also identified correlations between positive PD-L1 and improved survival [26, 27]. We could not identify the precise reason for better PFS in patients with positive PD-L1. Positive PD-L1 may represent immunogenic tumors that are sensitive to radiation-induced immunologic cell death [23].

One patient in this study developed radiation recall dermatitis. He was started on atezolizumab 1 month after RT. Three weeks later, he developed erythema over his left shoulder, which corresponded to the previous RT field. Dermatitis resolved completely with topical steroids. Radiation recall dermatitis occurs within a few days or weeks after the administration of immune check point inhibitors [28]. PD-1/PD-L1 inhibitors have been approved for patients with progressive urothelial carcinoma during or after chemotherapy based on phase II/III studies; therefore, the opportunities for the administration of anti-PD-1/PD-L1 antibodies have been increasing in patients with UTUC who received RT [5]. The exact pathogenesis of radiation recall dermatitis remains unknown, and severe cases have been reported [29, 30]. When administering immune checkpoint inhibitors in patients previously treated with RT, radiation recall dermatitis at previous RT fields should be monitored carefully, especially within a few weeks after administration.

## Conclusions

A standard treatment for recurrent or metastatic UTUC should be established through prospective, randomised studies; however, it is practically difficult to conduct such a study. We found that salvage and palliative RT was feasible and effective. There may be a survival benefit in patients with RT response with salvage or palliative RT. It is recommended to consider a higher dose with IMRT and concurrent CTx to improve the RT response. This study may be of help in selecting the optimal treatment option for patients with recurrent or metastatic UTUC. Further investigations of RT using advanced techniques and combining with systemic treatments are warranted.

## Data Availability

The datasets generated and/or analysed during the current study are not publicly available due to that individual privacy could be compromised, but are available from the corresponding author on reasonable request.

## Abbreviations

BED: Biologically effective dose
CCRT: Concurrent chemotherapy and radiotherapy
CR: Complete remission
CTV: Clinical target volume
GTV: Gross tumor volume
IMRT: Intensity modulated radiotherapy
OS: Overall survival
PD: Progressive disease
PFS: Progression-free survival
PR: Partial response
PTV: Planning target volume
RNU: Radical nephroureterectomy
SD: Stable disease
SPSS: Statistical Package for Social Sciences
TCGA: The Cancer Genome Atlas
UTUC: Upper tract urothelial carcinoma
